# Structural and Functional Neural Correlates of Treatment Response for Interpersonal Psychotherapy for Depressed Adolescents

**DOI:** 10.3390/jcm11071878

**Published:** 2022-03-28

**Authors:** Bonnie Klimes-Dougan, Zeynep Başgöze, Bryon Mueller, Andrea Wiglesworth, Kathrine A. Carosella, Melinda Westlund Schreiner, Ana Bortnova, Kristina Reigstad, Kathryn R. Cullen, Meredith Gunlicks-Stoessel

**Affiliations:** 1 Department of Psychology, University of Minnesota, Minneapolis, MN 55455, USA; wigle017@umn.edu (A.W.); caros006@umn.edu (K.A.C.); 2 Department of Psychiatry and Behavioral Sciences, University of Minnesota, Minneapolis, MN 55454, USA; bagze001@umn.edu (Z.B.); muell093@umn.edu (B.M.); reigstad@umn.edu (K.R.); rega0026@umn.edu (K.R.C.); mgunlick@umn.edu (M.G.-S.); 3 Department of Psychiatry, University of Utah, Salt Lake City, UT 84108, USA; mindy.westlund.schreiner@utah.edu; 4 Minnesota Department of Health and Human Services, Saint Paul, MN 55101, USA; anabortnova@gmail.com

**Keywords:** interpersonal psychotherapy, depression, adolescent, predictor, magnetic resonance imaging

## Abstract

Precision medicine approaches hold tremendous promise to advance current clinical practice by providing information about which individuals will benefit from which treatments. This pilot study evaluated if baseline structure and function of the salience and emotion brain regions implicated in adolescent depression, specifically the amygdala and anterior cingulate cortex (ACC), predict response to Interpersonal Psychotherapy for Depressed Adolescents (IPT-A). Adolescents (*n* = 15; mean age = 14.5 (1.6); 80.0% female) diagnosed with a depressive disorder completed brain scans before the start of a 16 week trial of IPT-A. Clinical measures assessing depressive symptoms were completed before, during, and after a trial of therapy. Results show that at baseline, greater ACC activation in the context of an emotion-matching task and greater amygdala-ACC resting-state functional connectivity was related to greater improvement in depression symptoms. There was minimal evidence that brain structure predicted changes in depressive symptoms. The present study is the first to evaluate neural predictors of IPT-A response. While the results are preliminary, these findings suggest some avenues for future research to pursue in the hopes that more will benefit from treatment.

## 1. Introduction

Depressive disorders impact about 16% of the population [1]. Depression is associated with impairment, chronic suffering, and early death [2]. Historical trends suggest that depression is on the rise and a leading cause of the global burden of disease worldwide [3]. Depression is commonly first evident during adolescence, and earlier onset is associated with a poorer prognosis, including premature death [4,5,6]. As adolescence is a sensitive period for both neurodevelopment and the onset of psychopathology [7], early intervention during this window may be particularly fruitful in mitigating the significant impacts of depression. Fortunately, evidence-based treatments are available and include psychopharmacological agents and psychotherapy [8,9]. However, work in this area is still needed given that 30 to 50% of the adolescents who receive care using the most well-validated treatments do not achieve remission [10,11]. Precision medicine approaches hold great promise for identifying which treatment(s) will be most effective for each individual [12], shortening the time it takes for an individual to receive the treatment that will ultimately bring them to remission.

Neuroimaging approaches have been shown to advance the understanding of treatment mechanisms and this line of work has the potential to guide individualized treatment. An accumulating body of imaging research implicates processing of negative affect for those with depression. The Salience and Emotion Network (SEN) is involved in detecting and regulating negative affect: the amygdala and other regions of the limbic system are involved in negative affect detection, while the anterior cingulate cortex (ACC) is an important structure that mediates the detection of negative affect and serves as a regulator of amygdala responses. There is a wealth of evidence showing that the SEN functions abnormally in depression with adults [13,14,15] and adolescents [16,17,18].

Relatively little research has been conducted on neural predictors of psychotherapy for those suffering from depression, particularly in adolescence, a period when connections within the SEN are changing rapidly [19]. Because a primary goal of psychotherapy is to teach strategies to both prevent and regulate negative emotions, psychotherapy may involve changes in synaptic plasticity by retraining of the implicit memory systems involved in negative affect [20,21,22]. Validated psychotherapy for adolescents with depression includes Interpersonal Psychotherapy (IPT) for Depressed Adolescents (IPT-A) [10,23,24]. IPT/IPT-A focuses on emotions in the context of the patient’s relationships. Conflict and stress in interpersonal relations are a common source of negative affect in adolescents with depression and are associated with the development, maintenance, and recurrence of depression [25,26,27]. IPT-A aims to decrease depressive symptoms by addressing interpersonal difficulties that arise from interpersonal role disputes, role transitions, interpersonal deficits, and loss. The interpersonal skills addressed in IPT-A include conflict negotiation, interpersonal problem solving, social approach/avoidance, response inhibition, and perspective-taking [23]. Successful early intervention through IPT-A may, in some ways, be related to the functioning of the SEN, as this network is strongly implicated in interpersonal relationships [28].

Prior research has yet to examine neural correlates or predictors of IPT-A with depressed adolescents or adults. Almost two decades ago, positron emission tomography (PET) scans were used to measure the change in regional cerebral glucose metabolism in adults with depression in the context of IPT (although the studies focused on brain mechanism but not predictors of IPT response) [29,30]. While we are not aware of subsequent research published on neural mechanisms or predictors of IPT/IPT-A, other forms of evidence-based psychotherapy that target negative emotions may yield some clues about possible predictors of treatment response. For example, research on neural predictors of Cognitive Behavior Therapy (CBT) may be relevant to consider, particularly research that examines volume, task activation, and resting-state functional connectivity (RSFC) of key nodes of the SEN. Regarding brain structure, there is accumulating evidence showing that larger ACC volume is related to a more favorable treatment response to CBT [31,32,33]. One of the only studies that focused on adolescents with depression considered RSFC of the amygdala and ACC as a whole-brain seed within the context of a trial of CBT group psychotherapy. It was found that higher baseline connectivity of both the amygdala and right subgenual ACC with the dorsolateral prefrontal cortex (DLPFC) was associated with larger improvements in depression scores [34], centrally implicating these key regions of the SEN. Research is not as consistent with regard to neural activation within the context of emotionally salient tasks, perhaps in part because a range of paradigms has been used. Ritchey and colleagues [35] found that greater activity of an area near the ACC in the context of viewing positive, negative, and neutral images was related to more improvement of depressive symptoms within the context of a trial for CBT in adults with depression. Rubin-Falcone and colleagues [36] failed to find that ACC or amygdala activation predicted CBT response using similar stimuli. Further, in a series of studies by Fu and colleagues [37,38] in which a viewing of sad faces task was used at baseline, greater ACC activation was a predictor of poorer CBT treatment response. Others have used paradigms in which negative words were viewed prior to the CBT trial. Interestingly, Siegle and colleagues [39] found that greater amygdala activation and less ACC activation predicted favorable treatment response, while Doerig and colleagues [40] found that greater amygdala activation was associated with a less favorable response to treatment. More work is needed, but these identified patterns provide some key evidence that the SEN may be a useful predictor of psychotherapy treatment response and may have implications for IPT-A.

While randomized trials are crucial to confirm neurobiological predictors of different treatment responses for personalization, an early step is to examine neural correlates of treatment response in the context of a validated treatment. In this current pilot project, we aimed to identify key neurobiological constructs that are associated with abatement of depressive symptoms within the context of a trial of IPT-A. We predicted that a greater ACC volume and greater amygdala-ACC RSFC at baseline would be associated with a better response to treatment. We also hypothesized that ACC and amygdala activation during a baseline administered task that probed negative emotion would predict greater improvement in depressive symptoms, although directional predictions were not made. The findings of this pilot study are preliminary and intended to be hypothesis-generating rather than confirmatory.

## 2. Materials and Methods

### 2.1. Participants

Adolescent participants (ages 12–16) were part of a larger study (N = 63) approved by the Institutional Review Board of the University of Minnesota using a Sequential Multiple Assignment Randomized Trial (SMART) to evaluate IPT-A [41]. Participants in the current study were those who opted into a more complete assessment in which neuroimaging, neuroendocrine, and cognitive measures were administered (hereafter referred to as BIO). The focus here is on the MRI evaluations. Of the 37 participants who consented to participate in the BIO study, 19 underwent baseline imaging; of these 18 usable scans 3 participants had not completed pre-post intervention clinical measures, leaving 15 participants with clinical and had sufficiently high-quality structural or functional MRI (fMRI) data to be included in the current study (*n* = 15 structural data; *n* = 14 RSFC data; *n* = 12 emotion-matching task data). Details on imaging exclusion criteria are provided below in fMRI Processing and Analysis. Table 1 describes the demographic and clinical characteristics of this sample.

Inclusion for the larger study was based on a consensus clinical diagnosis of depressive disorder from child and parent reports on the (a) Kiddie Schedule for Affective Disorders and Schizophrenia-Present and Lifetime Version (KSADS-PL); [42], (b) a Children’s Depression Rating Scale, Revised (CDRS; [43]) raw score greater than 35, as well as clinician’s rating on the Global Severity Index (<65). Exclusion criteria for the larger study included any neurological or medical condition that could impact treatment, psychotic disorder, bipolar disorder, substance abuse, obsessive-compulsive disorder, conduct disorder, eating disorder, pervasive developmental disorder, intellectual disability, a severe episode of depression with a CDRS raw score greater than 75, current risk for suicide, concurrent active treatment for another mental illness, previous non-response to an adequate trial of fluoxetine or IPT-A, and medication usage for a psychiatric condition other than attention-deficit/hyperactivity disorder. Non-English-speaking families and female adolescents who were pregnant, breastfeeding, or having unprotected sexual intercourse were also excluded from the study. Additional exclusion factors for the participants who opted into the BIO protocol discussed here included MRI scanning contraindications (e.g., claustrophobia, metal implants).

### 2.2. Procedure

Participants were recruited from the Minneapolis and St. Paul metro area via flyers, radio and newspaper advertisements, school referrals, and clinic referrals. All participants signed informed consent and/or assent. They received monetary compensation for their time for each assessment visit, but not for participating in the IPT-A intervention. Diagnostic interviews with the adolescent and their parent were conducted at the participants’ initial visit to determine depression status. On the second baseline visit, participants completed a set of self-report and behavioral measures, including the baseline MRI scan. Participants then began their trial of IPT-A with a series of additional visits around week 8 (W8) and week 16 (W16) when depressive symptoms were assessed. Additionally, some participants were randomly assigned to be assessed for an interim visit at week 4 (W4) according to the SMART trial, although clinical measures obtained during this assessment were not included in this study. Adolescents who were not making sufficient progress showing a reduction in depressive symptom were randomized to augmentation of medication (10 mg per day for the first week and 20 mg per day for the following weeks and up to 40 mg per day for those who didn’t respond) or additional 4 sessions of therapy sessions arm of the SMART trial [41].

### 2.3. Clinical Assessments

The presence or absence of a DSM-VI-TR Axis I disorder(s) [44] was confirmed by consensus meetings following a semi-structured clinical diagnostic interview. Adolescent participants and a legal guardian completed independent interviews with an evaluator using the KSADS-PL at baseline. The interviews were conducted by highly trained individuals such as clinical psychologists, child psychiatrists, or advanced trainees enrolled in clinical psychology doctoral programs under the direct supervision of a licensed clinician. For this study, we considered the baseline, W8, and W16 on two measures of depressive symptoms. One index of treatment response was the CDRS percent reduction from baseline. The CDRS is based on a semi-structured clinician interview with parents and adolescents that assesses 17 symptom areas related to current depression and is commonly used in treatment studies because it has been shown to be sensitive to change [45]. A secondary index of treatment response (percentage reduction from baseline) was the Beck Depression Inventory-II (BDI) [46], a well-validated, 21 item self-report measure of current depressive symptoms. In addition, raw differences from baseline for the CDRS and BDI scores (a measure that is not influenced by the initial level of symptoms) were considered as exploratory measures as they provide an alternative index that is based on the absolute number of symptoms.

### 2.4. Treatment

All BIO participants received individual IPT-A [23,24], an evidence-based, time-limited psychological intervention that aims to decrease depression symptoms by improving interpersonal functioning. It addresses one or more of four interpersonal problem areas: grief, role disputes, role transitions, and interpersonal deficits. IPT-A was administered for 12 sessions with standard, fifty-minute, outpatient individual therapy sessions within 16 weeks to account for holidays, absences, etc. As per the SMART trial study design of the larger sample, for the BIO, when participants were not responding, additional sessions of IPT-A or fluoxetine augmented the standard treatment regimens (for more details, see [41]).

### 2.5. Brain Imaging Acquisition

Every effort was made to scan all participants in the BIO with a 3T Siemens Trio MR system at the Center for Magnetic Resonance Research (CMRR) at the University of Minnesota at baseline. Structure: Structural scans were acquired using a four-minute T1-weighted MP-RAGE (Magnetization-Prepared Rapid Gradient-Echo) sequence with the following parameters: Repetition time (TR) = 2530 ms, echo time (TE) = 3.63 ms, inversion time (TI) = 1100 ms; 1 mm isotropic voxels, field of view (FOV) = 256 × 176 mm, flip angle = 7 degrees, parallel acquisition technique (PAT) = 2. Resting-State: fMRI scans were obtained using a whole-brain T2*-weighted functional acquisition with scan parameters of: TR = 2 s, TE = 30 ms, FOV = 220 mm × 220 mm, a 64 × 64 matrix, 34 slices acquired with a 4 mm thickness and no skip, 180 volumes, 6:04. Emotion-Matching Task: Task-based fMRI scans were obtained using a whole-brain T2*-weighted functional acquisition with scan parameters of: TR = 2 s, TE = 28 ms, FOV = 200 mm × 200 mm, a 64 × 64 matrix, 34 slices acquired with a 3 mm thickness and 1mm skip, 197 volumes, 6:38. Field Maps: Separate field map data was acquired using the Siemens field map sequence with a voxel parameter matching those of the fMRI task and rest acquisitions. Field map data were used to correct the fMRI data for the geometric distortion caused by magnetic field inhomogeneities.

### 2.6. MRI Emotion-Matching Task Description

The emotion-matching task is a widely used task to assess basic threat systems responses in which participants match either emotionally expressive faces (characterized by fear or anger) or neutral shapes (see [47] for more details on the task). The task was conducted via E-prime software [48], and stimuli were projected onto a screen inside the bore of the scanner. The negative emotion block consisted of black-and-white photographs of human faces depicting anger and fear (i.e., emotion condition) [49]. The control block consisted of black horizontal and vertical ellipses (i.e., shape condition). Participants were instructed to look at the picture in the top row and to select one of the two pictures in the bottom row that matched the emotion of the top row using a response box. The task consisted of 13 counterbalanced blocks, 24 s each: five face-matching blocks, five shape-matching blocks, and three blocks with only a fixation cross.

### 2.7. Brain Imaging Preprocessing

Preprocessing of the MP-RAGE T1-weighted scan was conducted using FreeSurfer (https://surfer.nmr.mgh.harvard.edu/, accessed on 18 March 2022). Structure. Amygdala volumes and ACC cortical thickness were calculated via FreeSurfer’s automated segmentation tools [50]. FreeSurfer output was visually inspected, particularly to make sure of the quality of the segmentation of grey matter around ACC, and no corrections were required. Therefore, (out of *n* = 15) no participant was excluded from further structural analyses. Resting State. Preprocessing of the RS fMRI data was conducted using FSL MELODIC (Multivariate Exploratory Linear Optimized Decomposition into Independent Components), which uses Independent Component Analysis (ICA) to decompose resting state fMRI data into spatially independent components, where each component is represented by a spatial map and a time course. Since we have a small sample of participants, we preferred to denoise the ICA components manually depending on their spatial map, time course, and power spectra. This allowed us to exclude all the components deemed as “noise” (most of them related to motion and some related to physiological signal such as heartbeat) and keep only the components deemed as “true signal”. We then then registered these denoised images to standard space. After extracting the time series for left and right amygdala using the Harvard-Oxford Subcortical Atlas [50] for each person we conducted two whole-brain regression analyses using FSL FEAT (FMRI Expert Analysis Tool) in which we measured the cross-correlation of timeseries between the “seed” (left and right amygdala) and each voxel of the brain was calculated and converted to z-scores. Then, we applied bilateral ACC masks (defined using Talairach Daemon labels [51]) to these whole-brain amygdala maps, to obtain average z-scores within those masks which represent connectivity between amygdala and ACC. Emotion-Matching Task. Preprocessing of the task fMRI data was conducted using FSL (www.fmrib.ox.ac.uk/fsl, accessed on 18 March 2022). To conduct a regression analysis measuring neural activation FSL FEAT was used. Two explanatory variables (emotion and shape conditions) were included, and the contrast of interest was emotion > shape. Volumes with a framewise displacement (FD) value > 0.5 mm were tagged and then included as a confounding factor in the FEAT analysis [52]. FSL Featquery was used to extract mean z scores within significant (z threshold = 3.1, corresponding to *p* threshold = 0.05) voxels from the 1st level results (z-statistic maps) for the emotion > shape contrast within regions of interest (ROI): Harvard-Oxford Subcortical Atlas for Amygdala [50] and Talairach Daemon labels [51] for ACC. These mean activation values from significant voxels from each ROI (bilaterally) were used in further analyses. Of note, different approaches were taken for the motion correction, because we believed this was more appropriate given the greater sensitivity to artificial correlations caused by motion in resting versus task fMRI data.

### 2.8. Statistical Analysis

All analyses were completed in SPSS version 25. The primary study aim was to examine the relation between baseline neurobiological mechanisms and treatment response. We conducted linear regression analyses between bilateral neurobiological structure (amygdala volumes, ACC cortical thickness), RSFC between amygdala and ACC, and activation towards the emotion > shape contrast for the emotion-matching task (amygdala and ACC), with change in depressive symptoms. That is, the primary clinical outcomes were based on percent reduction of BDI scores and CDRS scores. We conducted follow-up linear regression analyses to control for age, family income, and (yes/no) augmented medication effects independently. Follow-up exploratory analyses for difference scores for depressive symptoms were reported when significant. Finally, given that our small sample size provided limited statistical power, we did not correct for multiple comparisons, and presented the results as preliminary findings. We also considered patterns of responses using the following guidelines to determine the strength of effect size: 0.1 to 0.3 represent a small to medium effect, 0.3 to 0.5 represent a medium to large effect, and >0.5 represent a large to very large effect size [53].

## 3. Results

### 3.1. Clinical Results

The average number of IPT-A sessions attended by adolescent participants in this study was 11.16 (range = 7 to 16 sessions; mode = 12 sessions which is considered a standard dose of IPT-A, with all by two participants participating in at least 12 or more sessions). There were 8 different therapists who trained and implemented the IPT-A with the 15 adolescent participants in this study (with any one therapist following one to three of the study participants). CDRS and BDI scores were correlated at *r* = 0.57, *p* = 0.025 at baseline, *r* = 0.56, *p* = 0.029 at W8, and *r* = 0.77, *p* = 0.001 at W16. All of our participants met the criteria for significant clinical depressive symptoms based on the clinician-rated symptoms of parent and child reports at baseline (CDRS, see Table 1). On average, participants improved in their CDRS ratings by 19.6% by W8 and 29.3% by W16. Based on a threshold of a raw score of less than 28 on the CDRS, 6.7% of the participants remitted by W8 and 26.7% remitted by W16. At baseline, this sample was on average in the severe range of self-reported depression symptoms (BDI, see Table 1). Respectively by W8 and W16 of IPT-A, the adolescents improved on self-reported symptoms on the BDI by 43.4% and by 53.4% on average. Based on a raw score of less than 10 on the BDI, 33.3% remitted by W8 and 46.7% remitted by W16. Fluoxetine was added at W4 for four participants and at W8 for another four participants (as per the SMART trail study design of the larger study [41]). Where noted, subsequent results also account for medication status, whether fluoxetine was or was not augmented, as a covariate.

### 3.2. Brain Structure Results

As shown in Table 2, neither amygdala volume nor ACC cortical thickness measured at baseline was significantly associated with changes in depressive symptoms based on CDRS or BDI scores at W8 or W16 of IPT-A. When controlling for income, less left ACC cortical thickness was significantly associated to percent improvement in CDRS scores at W8 (*B* = −0.65, *p* = 0.023). No additional significant associations were noted when follow-up analyses were conducted controlling for age, income, or medication status.

### 3.3. Resting-State Results

As shown in Table 3, greater RSFC between the left amygdala and the ACC was significantly associated with percent improvement of BDI scores at W16 (*B* = 0.61, *p* = 0.020). These results remained significant when controlling for age (*p* = 0.034), income (*p* = 0.036), and medication status (*p* = 0.035) in follow-up linear regression models. When controlling for age, RSFC between the left amygdala and ACC was also significantly associated with CDRS percent improvement at W8 (*B* = 0.559, *p* = 0.049). No other significant associations were found between the right amygdala and ACC RSFC with the CDRS or BDI.

### 3.4. Emotion-Matching Task Activation Results

As shown in Table 4, greater right ACC activation during the Emotion-Matching task at baseline was significantly associated with percent symptom improvement of depressive symptoms based on the BDI at W8 (*B* = 0.62, *p* = 0.040). This effect remained significant in follow-up linear regression analyses that controlled for age (*p* = 0.034), income (*p* = 0.036), but not medication status (*p* = 0.055). No other associations were significant at the bivariate level. While right ACC activation at baseline was not significantly related to BDI improvement W16 at the bivariate level (*B* = 0.58, *p* = 0.060), we did identify a significant association when controlling for income in the linear regression model (*B* = 0.78, *p* = 0.037). Though non-significant, activation in the left amygdala demonstrated a large effect in the expected direction when predicting CDRS improvement at W8 (*B* = 0.59, *p* = 0.054), and was significantly associated with CDRS W8 improvement when controlling for age (*B* = 0.62, *p* = 0.022). Contrary to our hypotheses, right amygdala and left ACC activation to the emotion-matching task failed to show a significant association with the clinical outcome measures.

### 3.5. Follow-Up Analysis

We conducted analyses exploring if neural structure or function were related to raw score depressive symptom improvement. There were no significant findings with regard to the structure. Greater left amygdala to ACC RSFC was significantly associated with raw score improvement on the BDI at W16 (*B* = 0.57, *p* = 0.033). Greater left amygdala activation during the emotion-matching task was significantly associated with raw score improvement on the CDRS at W8 (*B* = 0.69, *p* = 0.020). This effect remained significant in follow-up regression analyses when controlling for age (*p* = 0.007) and medication (*p* = 0.036) but not when controlling for income (*p* = 0.057).

## 4. Discussion

There is an urgent need for new knowledge to guide the personalization and tailoring of treatment approaches to optimize clinical response outcomes. This process may be especially critical in the earliest stages of illness when there is an opportunity to divert significant worsening in clinical trajectories and restore health. Further, most of the existing research has been conducted with adults. Given the differences in brain development, the extent to which the work with adults identifying neural predictors of treatment response is relevant to adolescents when brain plasticity is heightened is unclear. In a recent review of the literature of predictors of treatment response for adolescents with depression, Ang and Pizzagalli [54] concluded that “Studies on biomarkers that truly reflect pathophysiology are scarce and difficult to draw conclusions from” (p. 18). Indeed, this line of work is just beginning to be undertaken. While the current work has the strength of a prospective design to evaluate neurobiological predictors of treatment response, given its small sample and single-arm design, the present study is intended to only serve as hypothesis generation for future studies evaluating neural predictors or moderators of psychotherapy response for adolescents with depression.

One of the consistently implicated regions in studies examining predictors of treatment response has been the ACC (e.g., [55]). Similarly, the most consistent results of the current study implicate the ACC as a predictor of change in depressive symptoms in the context of IPT-A. While our results failed to show consistent evidence of ACC structure as a predictor of treatment response, greater baseline activation of the right ACC in response to negative emotion stimuli was shown to predict a more favorable treatment response. This result was found across W8 and W16 outcomes for the BDI, an index based on child self-report. Of interest, this finding shows some similarities to work with adults [35], even if the broader literature shows a wide variation in the direction of results and the exact brain regions implicated (e.g., different hemispheres, subgenual ACC). It is possible that individuals with greater ACC activation at baseline have the capacity for greater change post-treatment, thereby driving this effect. A different perspective for how ACC is functioning may support regulatory capacity. In the current work, greater left amygdala-ACC RSFC also predicted greater improvement in depressive symptoms. Together these results suggest that ACC and the broader SEN may be important to examine when attempting to identify biomarkers that may predict treatment response.

Studies with adults that have incorporated neuroimaging into other types of psychotherapy have also implicated cingulate and limbic regions. Given that the ACC is a general predictor for a range of pharmacological and psychotherapeutic interventions, the extent to which the ACC will be a useful biomarker for differential prediction of IPT-A treatment response for those suffering from depression is unclear. An example of this type of work includes evidence in an adjacent brain region, also part of the SEN: Mayberg and colleagues have demonstrated that functioning of the insula can differentially predict response to psychopharmacological versus psychotherapeutic interventions [56], with lower activation of the anterior insula predicting a better response to CBT and higher activation of the anterior insula predicting a better response to SSRI. However, it is premature to discount the activation of the ACC to assign youth to treatment, for fully powered randomized control trials will be needed to make this determination.

In this study, results suggesting the predictive value of the amygdala were very scarce. The results failed to show evidence of amygdala volume as a predictor. In addition to the findings discussed above regarding connectivity, left amygdala activation during the emotion faces viewing task was associated with CDRS raw score improvement at W8 (for both percentage improvement and raw change scores). A similar pattern was only occasionally noted in psychotherapy studies with adults [39] but has also been found in adolescents treated with SSRIs [57]. While not a treatment trial, Canli and colleagues [58] found that greater amygdala activation to emotional faces predicts a greater decline in depressive symptoms over an eight-month period, controlling for medication status. Adjacent brain structures have also been shown to predict treatment response. Although not the focus of this study, based on other work [59], we explored hippocampal structure and activation during the emotion-matching task (Supplementary Materials), showing some tentative evidence that hippocampal volume may be a candidate for future consideration, as the emotional memories laid down when addressing interpersonal threats are critically important to the adaptive capacities of the individual. Future work should consider other brain networks likely to be implicated in IPT that were not examined here, including the default mode network and more distributed networks involving social processes, cognitive control network (e.g., [34]), and reward network which has been found to predict CBT treatment responses in adolescents [60] and Behavioral Activation Therapy in adults [61].

The Research Domain Criteria (RDoC) initiative [62] suggests that multiple-level approaches obtain the most informative outcomes. Including multiple units/levels of analysis allows examining relevant systems across the brain. This approach is increasingly used with adults [63]. This multilevel approach has recently been used with adolescents. For example, in an open-label study on youth with depression who were undergoing psychopharmacological treatment with SSRIs [57], in addition to neural activation and connectivity predictors, elevations of the hypothalamic pituitary axis were identified in those more likely to benefit from pharmacotherapy. While here we addressed multiple-levels of neural structure and function, this multilevel approach has yet to be routinely applied to psychotherapeutic interventions with depressed adolescents. Together, these findings point to the utility of taking a multiple index approach to assess treatment response.

There are significant study limitations. This work is exceptionally challenging to undertake given that in addition to the ongoing psychotherapy sessions, there is extensive coordination between parents and children’s schedules for the lengthy assessments conducted before, during and after treatment. Indeed, only about a third of the participants who initially consented were included in this analysis. This also resulted in a sample of convenience that was generally well resourced and represented limited racial or ethnic diversity. Additionally, due to issues related to subject motion and technical problems, the number of scans with usable data was smaller than expected. In the end, the exceptionally small sample size precluded any findings from being confirmatory; instead, they should be considered as possible avenues to explore in future work with considerably larger samples. Overall, models were simplistic and did not account for multiple comparisons. If avenues relevant to personalization are to be realized, future work will need to go beyond considering prediction of IPT-A treatment response and consider randomized control trials with multiple arms of assignments to different treatments. Future work may also apply machine learning approaches for algorithm development [64] and integration of clinical, cognitive, physiological, neural, and genetic data in prediction algorithms [65]. When more fully realized, this translational line of work holds enormous potential for advancing understanding of the pathophysiology of depression, promoting the development of more effective neurobiologically-informed interventions, and eventually aiding in the efforts to identify biological markers that can advance personalized treatment [12].

## 5. Conclusions

Precision medicine approaches hold considerable promise, yet research on adolescents experiencing depression is far from being fully realized. In this first study to examine neurobiological predictors of IPT-A, the pattern of findings highlights avenues worthy of further consideration. Personalized treatments or efforts to determine the optimal treatment(s) for adolescents suffering from depression based on their neurobiological profile could represent a significant advance for our field.

## Figures and Tables

**Table 1:** Sample Characteristics and Descriptive Data.

Sample Descriptives (*n* = 15)			
Demographic Descriptives		Clinical Descriptives	
Age, M (SD)	14.5 (1.6)	Principal Diagnosis	
Sex, *n* (%)		MDD	14 (93.3%)
Female	12 (80.0%)	DD NOS	1 (6.7%)
Male	3 (20.0%)	CDRS, M (SD)	
Race, *n* (%)		Baseline	51.1 (10.4)
White	13 (86.7%)	Week 8	41.3 (12.4)
AIAN	1 (6.7%)	Week 16	36.2 (11.0)
Multiracial	1 (6.7%)	BDI, M (SD)	
Family income, *n* (%) ^a^		Baseline	28.8 (10.0)
15,000–24,999	1 (6.7%)	Week 8	17.7 (12.6)
25,000–39,999	2 (13.3%)	Week 16	14.1 (11.5)
40,000–59,999	3 (20.0%)	Medication administration	
60,000–89,999	3 (20.0%)	Week 4	4 (26.7%)
90,000–179,999	2 (13.3%)	Week 8 ^b^	8 (53.3%)
Over 180,000	3 (20.0%)		

Note: ^a^ *n* = 14, 1 person responded “I don’t know”; ^b^ in addition to the four participants that were administered medication after the Week 4 assessment, four additional participants were administered medication after the Week 8 assessment, resulting in eight total participants taking medication; Abbreviations: AIAN = American Indian or Alaska Native, MDD = Major Depressive Disorder, DD NOS = Depressive Disorder not otherwise specified, CDRS = Children’s Depression Rating Scale, BDI = Beck Depression Inventory.

**Table 2:** Results (standardized Beta coefficients) of linear regression analyses modeling brain structure as a predictor of clinical outcomes.

Structure	Outcome Measures			
	BDI % Improvement Week 8	BDI % Improvement Week 16	CDRS % Improvement Week 8	CDRS % Improvement Week 16
L ACC	−0.219	−0.380	−0.428 ^a^	−0.036
R ACC	−0.239	−0.193	−0.210	−0.004
L AMYG	0.098	0.202	0.365	−0.063
R AMYG	0.030	0.065	0.312	−0.226

Note: ^a^ becomes significant when controlling for income. Abbreviations: R = right, L = left, ACC = cortical thickness of the anterior cingulate cortex, AMYG = amygdala volume, BDI = Beck Depression Inventory—II, CDRS = Child’s Depression Rating Scale—Revised.

**Table 3:** Results (standardized Beta coefficients) of linear regression analyses modeling resting state functional connectivity as a predictor of clinical outcomes.

Connectivity	Outcome Measures			
	BDI % Improvement Week 8	BDI % Improvement Week 16	CDRS % Improvement Week 8	CDRS % Improvement Week 16
R Amyg—ACC	−0.192	0.048	0.163	0.057
L Amyg—ACC	0.409	0.613 *^abc^	0.466 ^d^	0.176

Note: * significant *p* < 0.05, ^a^ still significant when controlling for age; ^b^ still significant when controlling for income; ^c^ still significant when controlling for medication, ^d^ becomes significant when controlling for age. Abbreviations: ACC = anterior cingulate cortex, AMYG = amygdala, L = left, R = right, BDI = Beck Depression Inventory—II, CDRS = Child’s Depression Rating Scale—Revised.

**Table 4:** Results (standardized Beta coefficients) of linear regression analyses modeling average brain activation within the context of the emotion-matching task as a predictor of clinical outcomes.

Activation (Mean)	Outcome Measures			
	BDI % Improvement Week 8	BDI % Improvement Week 16	CDRS % Improvement Week 8	CDRS % Improvement Week 16
ACC L	0.408	0.459	−0.189	0.449
ACC R	0.624 *^ab^	0.583 ^c^	0.186	0.411
AMYG L	0.381	0.282	0.594 ^d^	0.357
AMYG R	0.158	−0.063	0.210	0.069

Note: * significant *p* < 0.05, ^a^ still significant when controlling for age; ^b^ still significant when controlling for income; ^c^ becomes significant when controlling for income; ^d^ becomes significant when controlling for age. Abbreviations: ACC = anterior cingulate cortex, AMYG = amygdala, L = left, R = right, BDI = Beck Depression Inventory—II, CDRS = Child’s Depression Rating Scale—Revised.

## Data Availability

In this study, patient confidentiality prevents us from publicly sharing data.

## Supplementary Materials

The following supporting information can be downloaded at: https://www.mdpi.com/article/10.3390/jcm11071878/s1, while not the focus of this study, we explored hippocampal volume. At the bivariate level, greater left hippocampal volume was significantly associated with percent improvement for BDI scores at W8 (*r* = 0.58, *p* = 0.029), even when controlling for income (*T* = 2.37, *p* = 0.037) in a linear regression model. This association did not maintain statistical significance when controlling for the effects of age or medication (*p*’*s* < 0.10) and no other associations between hippocampal volume and clinical outcome measures emerged as significant.
