# Supplementary material for: Structural and Functional Neural Correlates of Treatment Response for Interpersonal Psychotherapy for Depressed Adolescents
Source: J Clin Med. 2022 Mar 28;11(7):1878. doi: 10.3390/jcm11071878 (PMC8999886; doi:10.3390/jcm11071878)
Supplement: Supplementary file 1 [file jcm-11-01878-s001.zip › jcm-1616794-supplementary.pdf]

While not the focus of this study, we explored hippocampal volume. At the bivariate level, greater left hippocampal volume was significantly associated with percent improvement for BDI scores at W8 ($r = 0.58$, $p = 0.029$), even when controlling for income ($T = 2.37$, $p = 0.037$) in a linear regression model. This association did not maintain statistical significance when controlling for the effects of age or medication ($p's < 0.10$) and no other associations between hippocampal volume and clinical outcome measures emerged as significant.
